# Integration of neurogenesis and angiogenesis models for constructing a neurovascular tissue

**DOI:** 10.1038/s41598-017-17411-0

**Published:** 2017-12-11

**Authors:** Hiroyuki Uwamori, Takuya Higuchi, Ken Arai, Ryo Sudo

**Affiliations:** 1 School of Integrated Design Engineering, Keio University, Yokohama, Japan; 2 Neuroprotection Research Laboratory, Departments of Radiology and Neurology, Massachusetts General Hospital and Harvard Medical School, Boston, USA; 3 Department of System Design Engineering, Keio University, Yokohama, Japan

## Abstract

Neurovascular unit (NVU) is a basic unit in the brain, including neurons, glial cells, blood vessels and extracellular matrix. This concept implies the importance of a three-dimensional (3D) culture model including these cell types for investigating brain functions. However, little is known about the construction of an *in vitro* 3D NVU model. In the present study, we aimed at constructing 3D neurovascular tissues by combining *in vitro* neurogenesis and angiogenesis models using a microfluidic platform, which is a critical step toward the NVU construction *in vitro*. Three gel conditions, which were fibrin gel, fibrin-Matrigel mixed gel and fibrin-hyaluronan mixed gel, were investigated to optimize the gel components in terms of neurogenesis and angiogenesis. First, fibrin-Matrigel mixed gel was found to promote neural stem cell (NSC) differentiation into neurons and neurite extension. In particular, 3D neural networks were constructed in 2–8 mg/ml fibrin-Matrigel mixed gel. Second, we found that capillary-like structures were also formed in the fibrin-Matrigel mixed gel by coculturing brain microvascular endothelial cells (BMECs) and human mesenchymal stem cells (MSCs). Finally, we combined both neural and vascular culture models and succeeded in constructing 3D neurovascular tissues with an optimized seeding condition of NSCs, BMECs and MSCs.

## Introduction

The brain consists of neural tissues including neurons and glial cells, blood vessels wrapped by smooth muscle cells or pericytes, and extracellular matrix (ECM). The brain cells interact with each other both structurally and functionally. For example, endothelial tight junctions form blood brain barrier (BBB) to control the transport of substances to the brain parenchyma, and it is known that astrocytes and pericytes around blood vessels contribute to regulation of BBB functions^1,2^. This brain-specific structure is now well-known as the concept of neurovascular unit (NVU), which is a basic unit in the brain. In the field of brain tissue engineering, many studies have been conducted using single cell type, e.g. either neurons or glial cells, to simplify an experimental model. However, as the concept of NVU has been proposed, it becomes increasingly important to investigate interactions among brain cells for further understanding of brain functions and developing therapeutics of brain disorders. Therefore, it is necessary to construct a brain tissue with multiple cell types *in vitro*, which allows us to analyze the interactions of brain cells in NVU.

*In vitro* NVU culture models have been reported to recapitulate brain specific functions^3^. Especially, a Transwell assay has been used to analyze the function of brain endothelial cells (ECs) by coculturing ECs and astrocytes or pericytes^2,4^. These studies revealed that the expression of tight junction proteins increased in ECs when they were cocultured with astrocytes or pericytes. However, ECs were cultured two-dimensionally on a plastic dish or a cell culture insert, which are much stiffer than the ECM substrate exposed to cells *in vivo*. The difference in cell configuration and substrate stiffness may cause a different morphogenesis of ECs in two-dimensional (2D) culture compared to those in physiological conditions. To overcome the gap between 2D culture and *in vivo* conditions, it is required to develop a three-dimensional (3D) culture model where neural and vascular networks can be constructed in an ECM hydrogel.

Previous *in vitro* NVU culture models mimicked a specific structure of NVU in which an endothelial monolayer was in close proximity with astrocytes or pericytes^4,5^. In these models, differentiated glial cells or pericytes were used to construct such a specific structure. However, ECs formed a monolayer in these models while ECs form vascular structures *in vivo*. To construct vascular structures, ECs are required to be cultured in 3D hydrogel to mimic angiogenesis, which is a multi-step morphogenesis of ECs and mural cells. Such a 3D hydrogel also provides a scaffold for neural cells to mimic neurogenesis, which is also a multi-step process including neural differentiation from neural stem cells (NSCs) and neurite extension. Construction of a neurovascular tissue is realized through these processes, suggesting that the combination of *in vitro* neurogenesis and angiogenesis models is a critical step toward the NVU construction *in vitro*.

In this study, we present a 3D culture model in a microfluidic platform that allows us to construct neurovascular tissues by triculture of human NSCs, human brain microvascular ECs (BMECs) and human mesenchymal stem cells (MSCs). As a first step, we focused on constructing neurovascular tissues including three-dimensionally extensive neurons and capillaries, because construction of neurovascular tissues is essential for the next step to construct a 3D NVU structure including neurons, capillaries and glial cells. In the present study, cells were cultured in ECM hydrogel composed of fibrin, Matrigel and hyaluronan. The effect of the gel components on both neurogenesis and angiogenesis was investigated to construct neurovascular tissues. We also controlled the timing of cell seeding and cell distribution to optimize cell–cell and cell–ECM interactions in the process of neurogenesis and angiogenesis. In terms of angiogenesis, our previous study demonstrated that MSCs promoted vascular formation of human umbilical vein endothelial cells (HUVECs) and differentiated into α-smooth muscle actin (α-SMA)-positive perivascular cells^6^. These MSC-derived pericyte-like cells sparsely wrapped constructed vasculatures, which had continuous luminal structures. Based on these results, we hypothesized that BMECs could also construct vascular structures in BMEC-MSC coculture. The aim of this study was to investigate 3D microenvironments for culturing NSCs and BMECs with induction of neurogenesis and angiogenesis in a microfluidic platform, which is the critical step toward the development of an *in vitro* NVU model.

## Results

### Neurite formation of neurons differentiated from NSCs in different gel scaffolds

**Figure 1:**
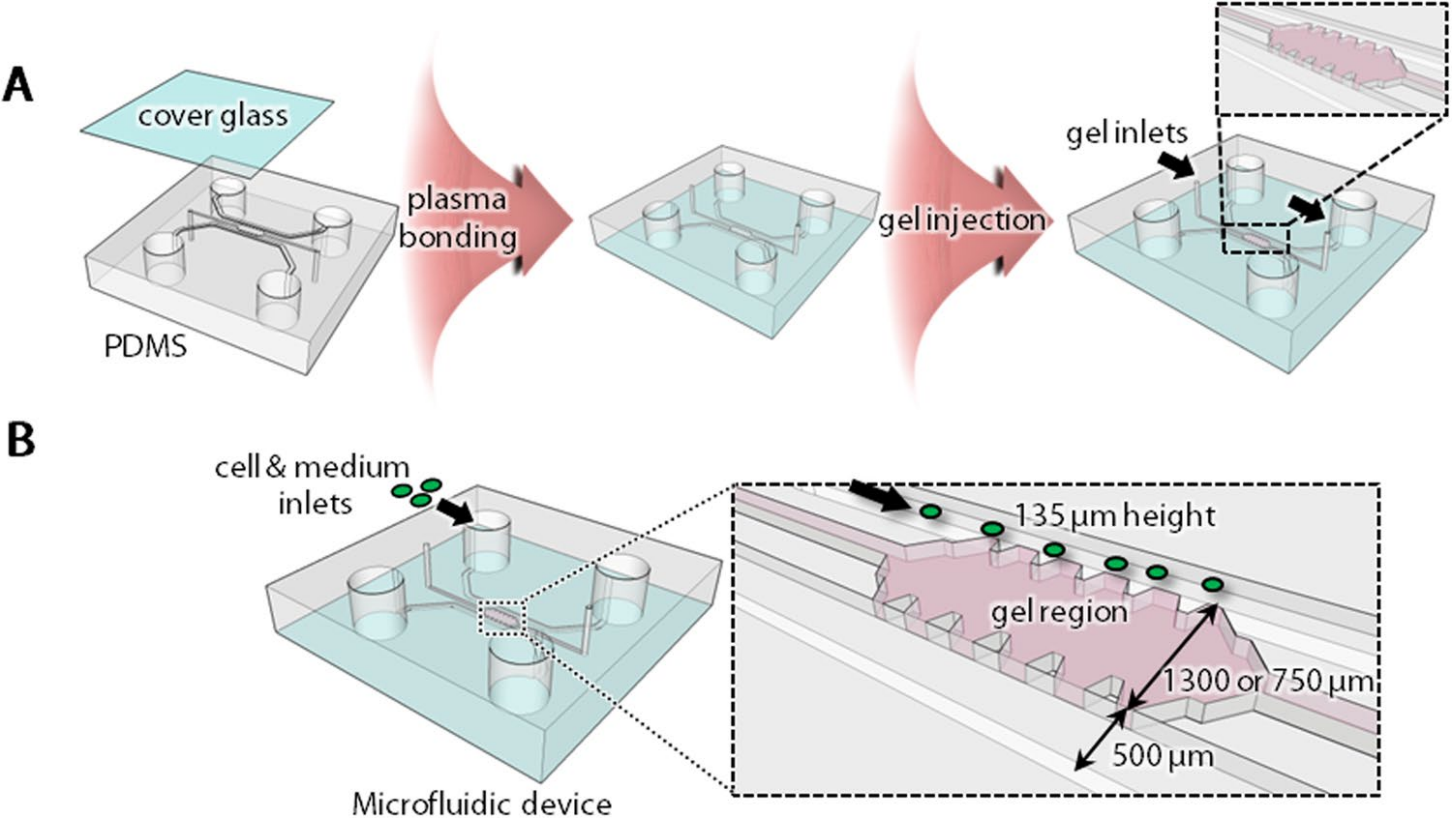
Schematic illustrations of a microfluidic device. (**A**) A PDMS device copied by an SU-8 mold was plasma-bonded with a coverglass to form microfluidic channels. Hydrogel pre-polymer was then injected into the central channel from two gel inlets (arrows). (**B**) There are two parallel microchannels separated by the gel region. BMECs and NSCs were injected into one of the microchannels (arrows).

**Figure 2:**
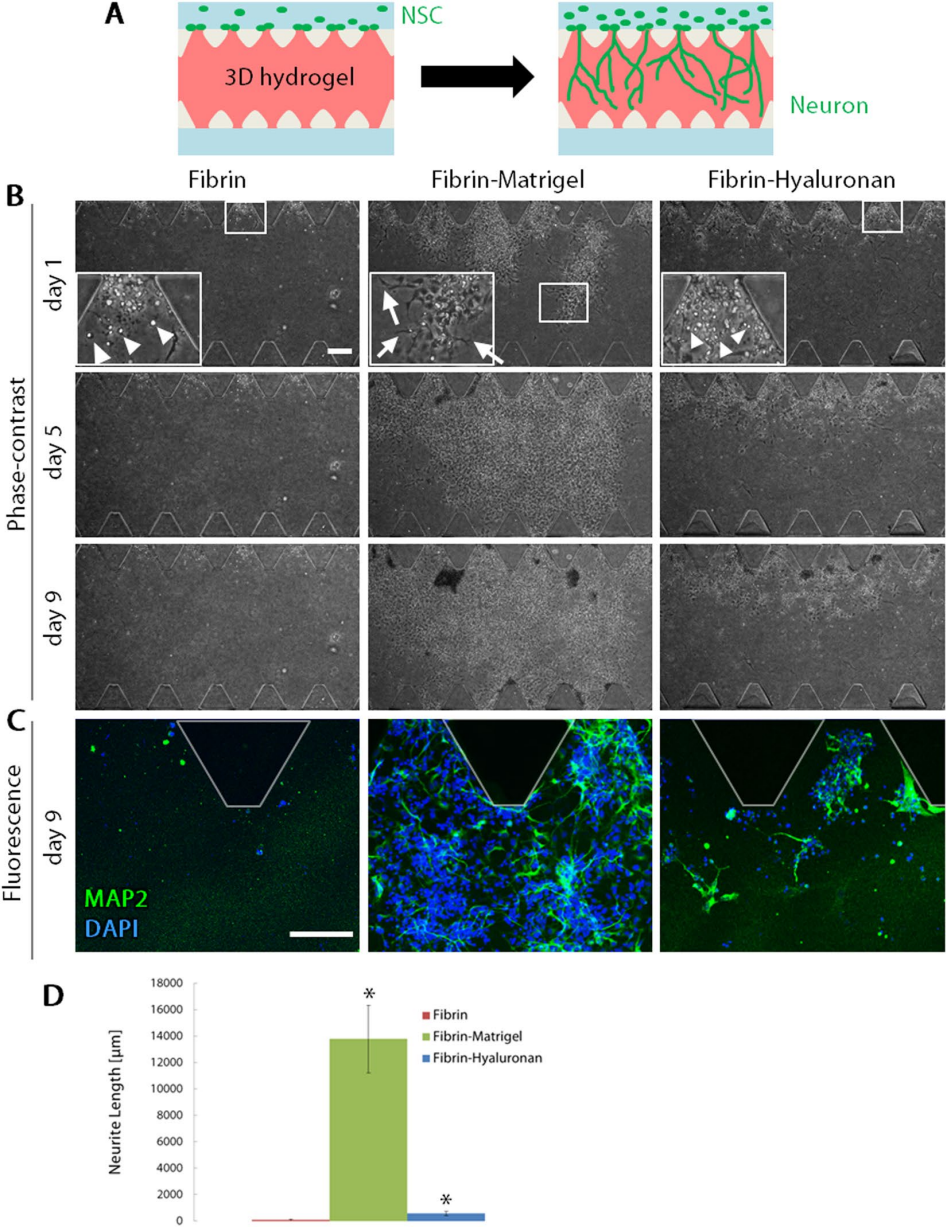
Neurite formation of NSC-derived neurons in different gel scaffolds. (**A**) Schematic illustrations of NSC culture for constructing a neural network. (**B**) Representative phase-contrast images of NSCs cultured in three different gel scaffolds; fibrin (left), fibrin-Matrigel mixed gel (middle) and fibrin-hyaluronan mixed gel (right). Arrowheads in insets indicate a round shape of NSCs (Fibrin, Fibrin-Hyaluronan), while arrows in an inset indicate an extended NSCs (Fibrin-Matrigel). (**C**) Representative immunofluorescence images of neurons (MAP2, green) and nuclei (DAPI, blue) on day 9. (**D**) Quantitative analysis of neurite length. Data are shown as the mean ± s.e.m. (N = 3, n ≥ 16). *p < 0.05 vs. Fibrin. Scale bars, 100 µm.

First, we cultured NSCs in a microfluidic device, which includes a 3D gel region (Fig. 1). In the NSC monoculture, we focused on the optimization of gel scaffolds for neurite formation (Fig. 2A). NSCs were cultured in different gel compositions including fibrin, Matrigel and hyaluronan. Fibrin gel has been commonly used for 3D cell culture because of its structural stability. Matrigel and hyaluronan are often used for the culture of neural cells because brain ECM is composed of laminin, hyaluronan and other proteins such as chondroitin sulfate proteoglycans^7^. Laminin is the main component of Matrigel. Although we tried to form a gel scaffold of 2 mg/ml Matrigel alone or 2 mg/ml hyaluronan alone, it was difficult to form these gels (data not shown). Therefore, we used fibrin as a basal gel scaffold and added Matrigel or hyaluronan, resulting in three gel conditions; fibrin gel, fibrin-Matrigel mixed gel and fibrin-hyaluronan mixed gel. NSCs were cultured in these gel conditions and cell morphology and migration were observed everyday by phase-contrast microscopy until day 9. The result showed that, in fibrin gel conditions, some cells migrated into the gel on day 1 (Fig. 2B, Fibrin). These migrating cells exhibited a round-shape without neurite extension (e.g., arrowheads in Fig. 2B, Fibrin, day 1, inset) and the amount of migrating cells did not increased until day 9. Similarly, NSCs exhibited a round-shape when cultured in fibrin-hyaluronan mixed gel (arrowheads, Fig. 2B, Fibrin-Hyaluronan, day 1, inset). The amount of migrating cells gradually increased until day 9. On the other hand, the morphology of NSCs was different in fibrin-Matrigel mixed gel; cells were extended within the gel (arrowheads, Fig. 2B, Fibrin-Matrigel, day 1, inset). In addition, migration of NSCs was the greatest in the gel.

To further investigate cellular morphology and differentiation of NSCs into neurons, immunofluorescence staining of cells on day 9 was performed for a neurons marker, MAP2. In fibrin gel condition, a few round-shape cells were positive for MAP2 but neurite extension was not observed (Fig. 2C, Fibrin). In fibrin-hyaluronan mixed gel, some NSCs were positive for MAP2 and exhibited short neurite-like structures (Fig. 2C, Fibrin-Hyaluronan). On the other hand, many NSCs in fibrin-Matrigel mixed gel were positive for MAP2 and extended neurites (Fig. 2C, Fibrin-Matrigel). Quantitative analysis of neurite length on day 9 revealed that neurite extension was significantly promoted in fibrin-Matrigel mixed gel (Fig. 2D).

### Formation of capillary-like structure in BMEC-MSC coculture in different gel scaffolds

**Figure 3:**
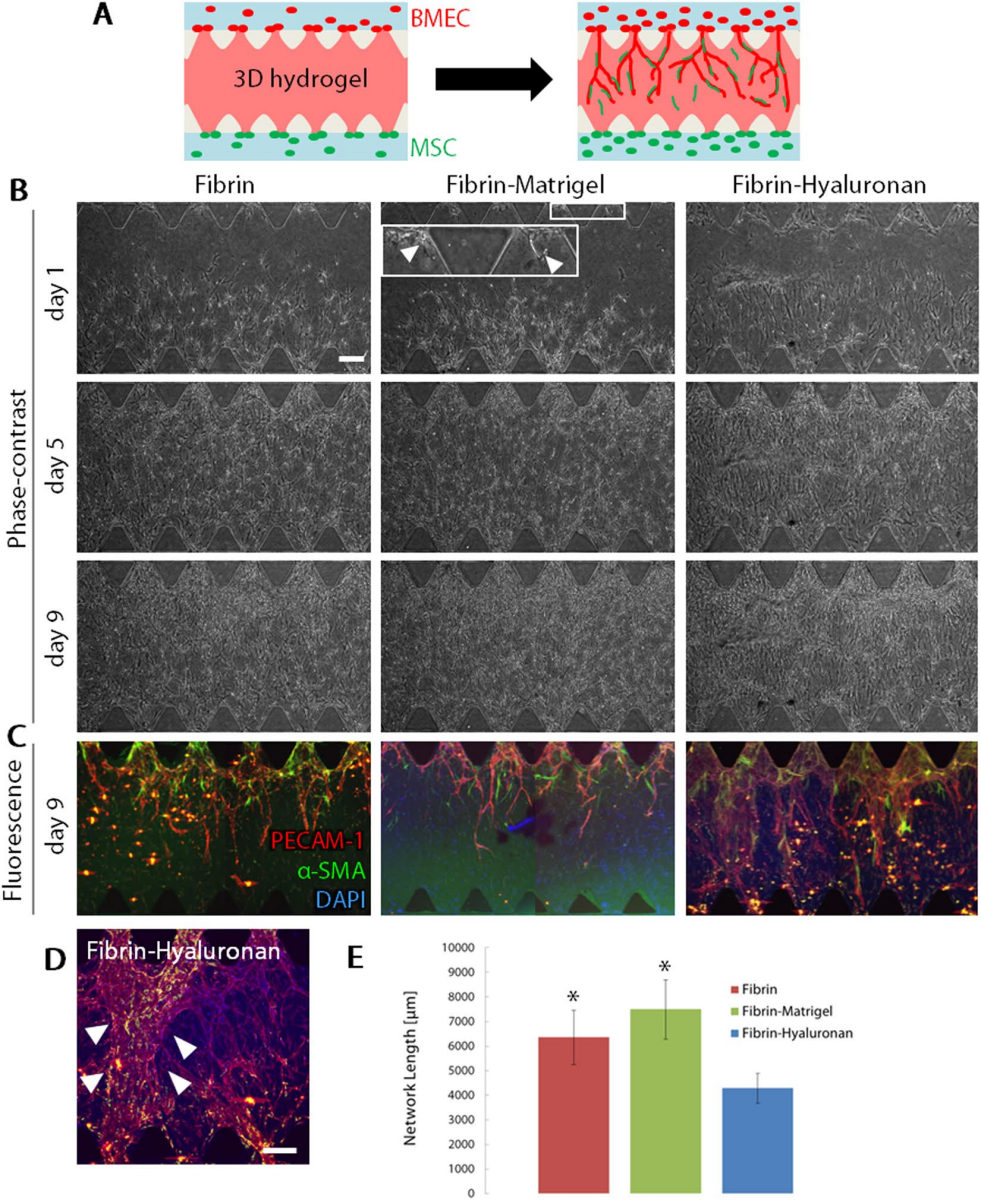
Formation of capillary-like structures in BMEC-MSC coculture in fibrin, fibrin-Matrigel mixed gel, and fibrin-hyaluronan mixed gel. (**A**) Schematic illustrations of BMEC-MSC coculture. (**B**,**C**) Representative phase-contrast images on days 1, 5 and 9, and corresponding immunofluorescence images of BMECs (PECAM-1, red), pericytes (α-SMA, green), and nuclei (DAPI, blue) on day 9. Arrowheads in an inset indicates vascular sprouts (Fibrin-Matrigel, day 1). Scale bar, 200 µm. (**D**) An enlarged image of cells in the fibrin-hyaluronan mixed gel. Arrowheads indicate a sheet-like structure of BMECs. Scale bar, 200 µm. (**E**) Quantitative analysis of network length. Data are shown as the mean ± s.e.m. (N = 3, n ≥ 9). *p < 0.05 vs. Fibrin-Hyaluronan.

Next, we tested the same gel conditions, which were fibrin gel, fibrin-Matrigel mixed gel and fibrin-hyaluronan mixed gel as tested for NSCs, in terms of capillary formation. Coculture of BMECs and MSCs was performed in these gel conditions. First, BMECs and MSCs were cultured in fibrin-Matrigel mixed gel to investigate whether capillary networks can be formed in the gel condition. BMECs were seeded into one channel while MSCs were seeded into the other channel (Fig. 3A). On day 1, MSCs dominantly migrated into the 3D hydrogel toward BMECs, while some BMECs formed vascular sprouts (arrowheads, Fig. 3B, Fibrin-Matrigel, day 1, inset). Both cell types increasingly migrated into the gel region until day 9. Because it was difficult to distinguish between BMECs and MSCs by phase-contrast microscopy, immunofluorescence staining was performed for PECAM-1 and α-SMA, which are markers for BMECs and perivascular cells, respectively. The immunofluorescence image revealed that BMECs formed vascular sprouts and constructed branching capillary-like structures while MSCs differentiated into α-SMA-positive perivascular cells on day 9. Similar capillary-like structures were also formed in fibrin gel, while sheet-like structure was observed in fibrin-hyaluronan mixed gel (Fig. 3C, Fibrin, Fibrin-Hyaluronan, Fig. 3D). Quantitative analysis of network length on day 9 revealed that construction of capillary-like structures was the greatest in fibrin-Matrigel mixed gel (Fig. 3E). In addition, we succeeded to construct capillary-like structures in fibrin-Matrigel mixed gel by culturing BMECs and MSCs in the same microchannel (Supplementary Fig. S1).

### Optimization of the concentration of fibrin-Matrigel mixed gel

**Figure 4:**
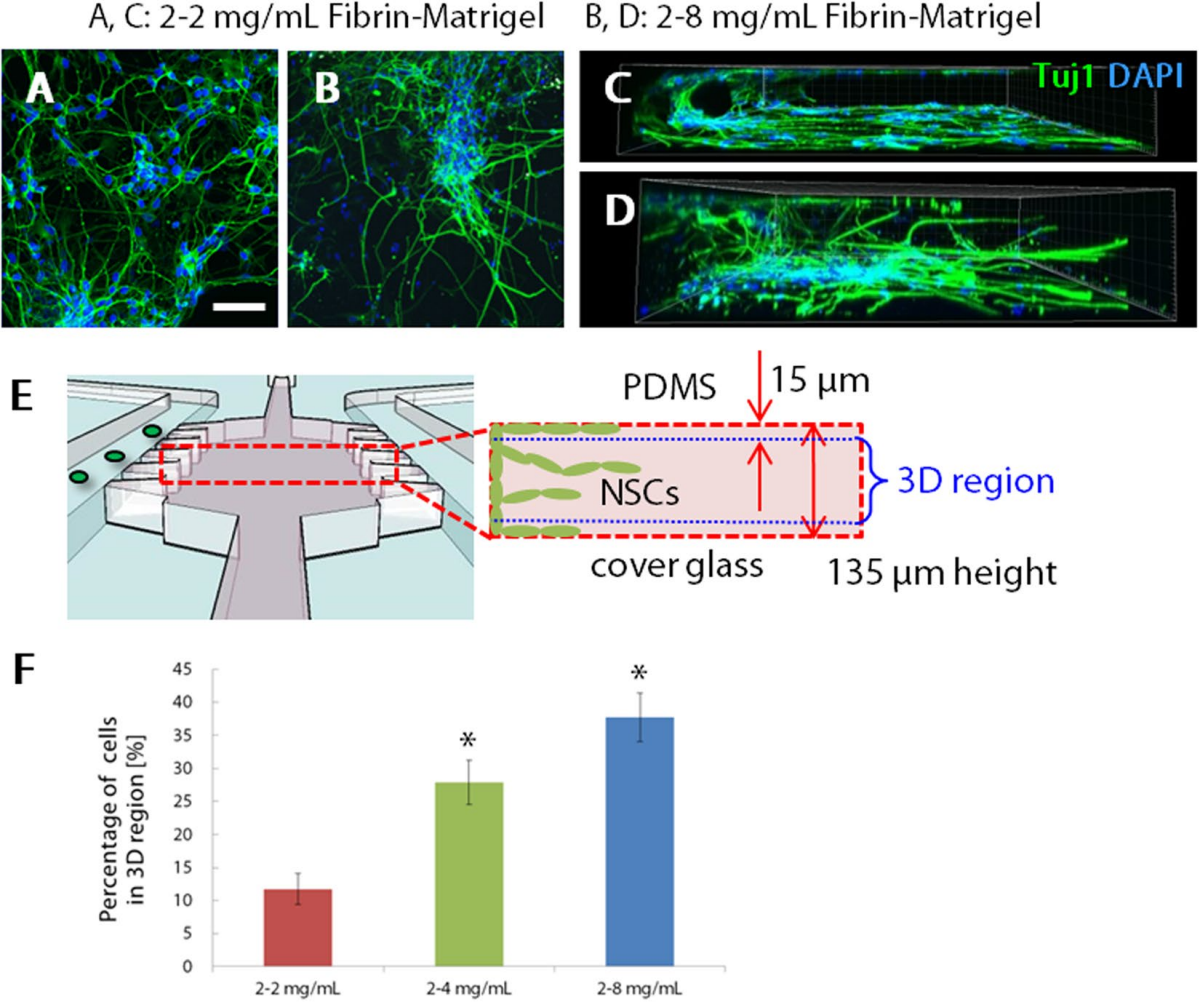
Optimization of the concentration of fibrin-Matrigel mixed gel for 3D migration of neurons. (**A**–**D**) Immunofluorescence projection images and corresponding 3D views of neurons in 2–2 mg/ml and 2–8 mg/ml fibrin-Matrigel mixed gel, respectively. Cells were fixed on day 21 and stained for neurons (Tuj1, green) and nuclei (DAPI, blue). Scale bar, 100 µm. (**E**–**F**) Quantitative analysis of the percentage of migrating cells in a 3D manner. The number of NSCs migrating into the 3D gel region. Data are shown as the mean ± s.e.m. (N = 3, n ≥ 12). *p < 0.05 vs. 2–2 mg/ml.

Based on the experimental results above, we used fibrin-Matrigel mixed gel for subsequent experiments. Since neurons extend three-dimensionally in the brain tissue, we investigated 3D distribution of NSCs in the gel. Confocal microscope images revealed that Tuj1-positive neurons derived from NSCs migrated into the gel region in a 2D manner along with the top and bottom surface of the gel region (Fig. 4A,C). As shown in Fig. 2, fibrin-Matrigel mixed gel promoted neurite extension more than fibrin gel, which suggested that Matrigel promoted neurite extension. Therefore, we fixed fibrin concentration as 2 mg/ml and increased Matrigel concentration. Specifically, Matrigel concentration was increased to be 4 or 8 mg/ml to form more stable gel structure. Consequently, we succeeded in inducing 3D neurite extension when NSCs were cultured in the modified gel with higher Matrigel concentrations (Fig. 4B,D). To quantify this 3D distribution of NSCs, the percentage of the number of cell nuclei in a 3D region against the entire gel region was calculated (Fig. 4E). The number of cell nuclei in the 3D region significantly increased as Matrigel concentration increased (Fig. 4F). In particular, the greatest 3D distribution was obtained in 2–8 mg/ml fibrin-Matrigel mixed gel, and the percentage was 37.7 ± 3.66%.

### The process of neurogenesis and neurite extension in NSC culture

**Figure 5:**
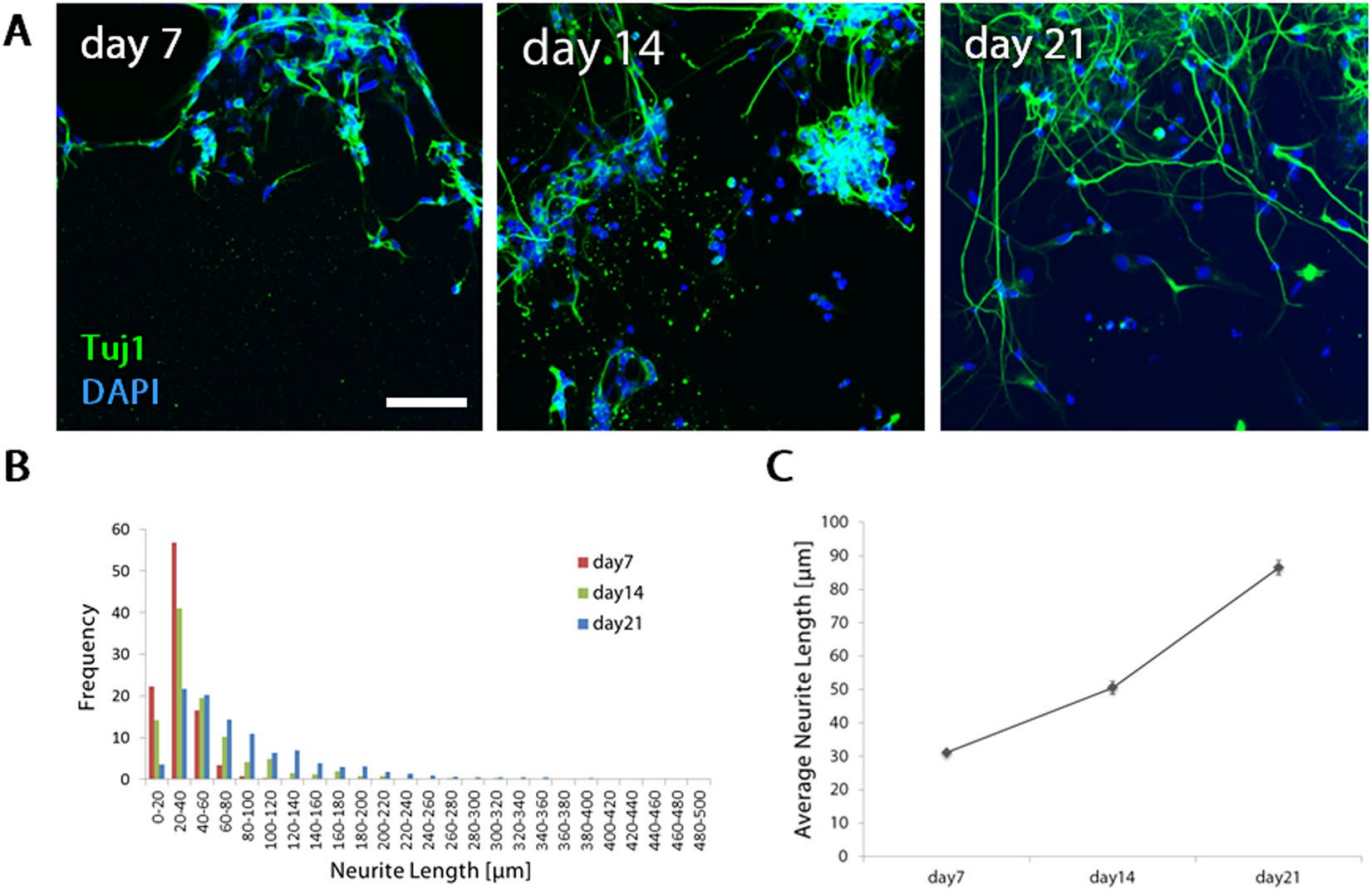
The process of neurogenesis in 21-day culture. (**A**) Immunofluorescence projection images of NSC-derived neurons in 2–8 mg/mL fibrin-Matrigel mixed gel. Cells were fixed on day 21 and stained for neurons (Tuj1, green) and nuclei (DAPI, blue). Scale bar, 100 µm. (**B**) A histogram of neurite length on days 7, 14, and 21. (**C**) Time course of average neurite length in 21-day culture. Data are shown as the mean ± s.e.m. (N = 3, n ≥ 12).

To further investigate differentiation of NSCs into neurons and the process of neurite extension, NSCs cultured in 2–8 mg/ml fibrin-Matrigel mixed gel were fixed on days 7, 14 and 21, and immunofluorescence staining was performed for neurons (Tuj1). Representative projection images showed that cells on day 7 were positive for Tuj1 but exhibited only short processes (Fig. 5A, day 7). However, cells on day 14 extended neurites, which further extended to form long neurites on day 21 (Fig. 5A, day 14, 21). According to the histogram of neurite length, the distribution of the neurite length of neurons shifted towards longer area as they were cultured for 7, 14 and 21 days, which suggested that neurons continuously elongated their neurites until day 21 (Fig. 5B). The longest neurite length was 139 μm, 300 μm and 445 μm on day 7, 14 and 21, respectively. The average neurite length increased in proportion to the culture days (Fig. 5C). The average neurite length was 31 ± 0.6 μm, 51 ± 2.0 μm and 86 ± 2.3 μm on day 7, 14 and 21, respectively.

### Triculture of NSCs, BMECs and MSCs for constructing neurovascular tissues

Finally, we combined the NSC culture model, which recapitulates neurogenesis, and BMEC-MSC coculture model, which recapitulates angiogenesis, for constructing neurovascular tissues. In the triculture, we seeded BMECs and MSCs in one microchannel and seeded NSCs in the other microchannel to separate angiogenesis and neurogenesis. When culturing BMECs and MSCs, there are two ways of cell seeding; seeding both cell types into one microchannel, or seeding BMECs into one microchannel and seeding MSCs into the other channel. We confirmed that capillary-like structures were constructed in both conditions (Supplementary Fig. S1). We performed three conditions in terms of the timing of cell seeding; “same-day seeding”, “BMEC-MSC pre-seeding”, and “NSC pre-seeding”. In the same-day seeding condition, BMECs-MSCs and NSCs were seeded on the same day. In the BMEC-MSC pre-seeding condition, BMECs and MSCs were seeded in the beginning of the culture, and NSCs were added on day 7 after vascular structures were constructed by BMECs and MSCs. In the NSC pre-seeding condition, NSCs were seeded in the beginning of the culture, and BMECs and MSCs were added on day 7 after NSCs migrated into the gel region and extended neurites.

**Figure 6:**
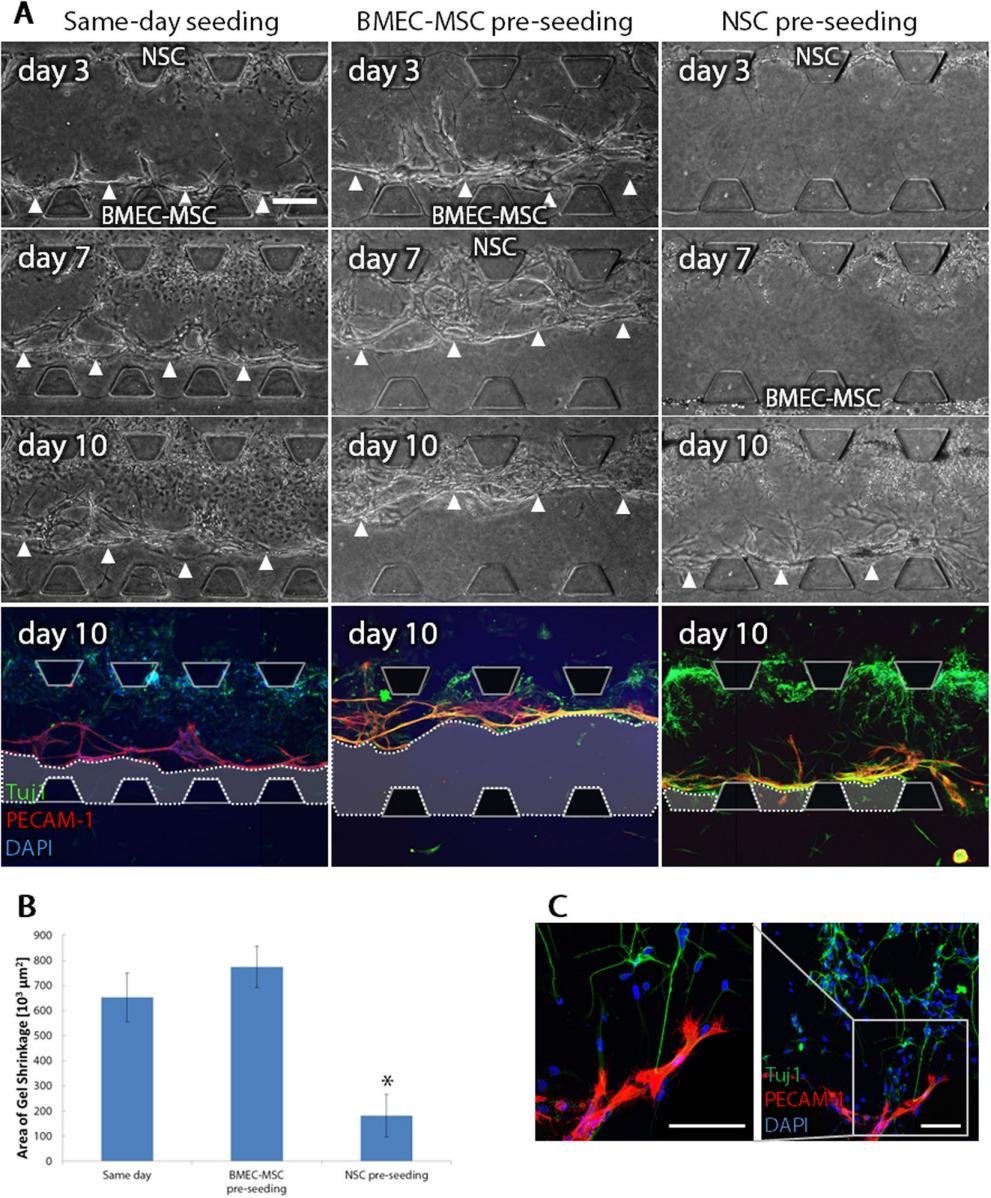
Triculture of NSCs, BMECs and MSCs for constructing neurovascular tissues. (**A**) Representative phase-contrast images on days 3, 7 and 10, and corresponding immunofluorescence images of neurons (Tuj1, green), BMECs (PECAM-1, red) and cell nuclei (DAPI, blue) on day 10. Arrowheads and areas surrounded by dotted lines in immunofluorescence images represent the gel shrinkage. Scale bar, 200 μm. (**B**) Quantitative analysis of the gel shrinkage in different seeding conditions on day 10. Data are the means ± s.e.m. (N = 3, n ≥ 9). *p < 0.05 vs. “Same day” and “BMEC-MSC pre-seeding”. (**C**) Enlarged views of neurons and BMECs in NSC pre-seeding condition. Scale bars, 100 μm.

In the same-day seeding condition, NSCs migrated into the gel region from the beginning of the triculture while BMECs started sprouting into the gel region. In the BMEC-MSC pre-seeding condition, BMECs also started sprouting into the gel region from the beginning of the culture, and some of capillary-like structures reached the end of the gel region, which is 750 μm far from the seeding point. However, in these seeding conditions, the gel structure shrank as vascular sprouts elongated into the gel region with increasing culture time (Fig. 6A, Same-day seeding, BMEC-MSC pre-seeding). Therefore, enough space was not maintained for NSCs to migrate and expand in the shrunk gel structure. On the other hand, in the NSC pre-seeding condition, NSCs migrated into the gel region before seeding BMECs and MSCs. NSCs differentiated into neurons and elongated neurite structures while capillary-like structures were constructed inside the same gel region after seeding BMECs and MSCs on day 7 (Fig. 6A, NSC pre-seeding). The area of the gel shrinkage was quantified on day 10 of the triculture (Fig. 6B). Gel shrinkage was significantly reduced in the NSC pre-seeding condition compared to the other conditions. In addition, enlarged view of neurons and BMECs in the NSC pre-seeding condition showed that capillary-like structures and neurites contacted to each other in the gel region (Fig. 6C). The constructed neurovascular tissues maintained at least for 10 days.

## Discussion

In the present study, we succeeded to construct 3D neurovascular tissues in ECM hydrogel by combining 3D angiogenesis and neurogenesis models. In the field of tissue engineering, it has been a big challenge to construct a 3D NVU structure including neurons, glial cells, and blood vessels. Previous studies reported *in vitro* NVU models using Transwell or microfluidic devices that were composed of ECs with/without astrocytes and pericytes to investigate BBB functions of ECs^2,8–11^. These culture models enabled us to investigate the functional characteristics of ECs such as endothelial permeability. However, cells in these models were cultured as endothelial monolayers, which is different from *in vivo* conditions.

Culturing brain cells in ECM hydrogel, which is closer to physiological conditions than a plastic substrate such as well plates, will lead to recapitulate more physiologically relevant morphogenesis. In addition, ECs or other kinds of brain cells adhering on a flat surface such as an insert membrane and a plastic dish have limited ability to realize specific structures or functions because of the limitation of signaling pathways, while brain cells in physiological conditions can interact with each other through various pathways such as paracrine and juxtacrine signalings. Brain cells in the Transwell model cannot interact with each other in the juxtacrine manner because of the physical compartment, while astrocytes and pericytes wrap around blood vessels through the basement membrane in a physiological condition^12^. This limitation of signaling pathway can cause a different morphology of cultured cells compared to those in physiological conditions. The stiffness of a plastic surface or coated ECM proteins on which cells adhere can also affect the morphology of these cells and differentiation of stem cells^13,14^. To overcome such limitations in previous 2D NVU models, our model provided a 3D neurovascular tissue that is constructed by inducing neurogenesis and angiogenesis in a microfluidic device. Our success in recapitulating neurogenesis and angiogenesis suggests that our microfluidic culture model provided a proper microenvironment for both neural and vascular cells to survive and function.

First we cultured NSCs in fibrin gel, fibrin-Matrigel mixed gel and fibrin-hyaluronan mixed gel. Some NSCs cultured in fibrin gel migrated in the gel region. However, the number of migrating cells did not increase and the neurite extension was not confirmed. When NSCs were cultured in fibrin-hyaluronan mixed gel, the number of migrating cells gradually increased until day 9, and some cells elongated neurites. Therefore, these data suggested that hyaluronan has an ability to promote NSC proliferation, migration and neurite extension. However, the proliferation of NSCs and the neurite extension in fibrin-Matrigel were even promoted compared to the other conditions. It is known that various factors can promote or inhibit neurite extension. A composition of ECM proteins is one of the important factors in regulating neurite extension. Previous studies showed that laminin can guide neurite extension and promote migration of growth cone of neurons^15,16^. Neurons bind to laminin, which is the main component of Matrigel, through β1 integrin^17,18^. A previous report showed that axons of neurons which bound to laminin expressed a β1 integrin, and laminin/β1 integrin signaling contributed to axon development and promoted axon elongation^17^. Similarly, in the present experiments, it is suggested that NSC-derived neurons, which were cultured in fibrin-Matrigel mixed gel, bound to laminin and constructed 3D neural networks through the laminin/β1 integrin signaling.

Three-dimensional neurite extension is one of the important achievements in this study. When NSCs were cultured in 2–2 mg/ml fibrin-Matrigel mixed gel, neurons derived from NSCs elongated neurites but the constructed neural networks were two-dimensionally distributed along the bottom glass surface. We hypothesized that this 2D neurite extension was induced due to the sparse gel fiber networks arising from the low concentration of the mixed gel. Therefore, we modified the gel concentration to be 2–8 mg/ml fibrin-Matrigel mixed gel for increasing the density of the gel fiber networks, resulting in a success in the construction of 3D neurite networks. Although we increased Matrigel concentration, we did not increase the fibrin concentration because we confirmed that fibrin gel did not promote neurite extension.

In our neurovascular tissue culture model, NSCs differentiated into neurons and they continued elongating neurites at least for 21 days. Average neurite length increased gradually with culture time, indicating that neurons can elongate their neurites even longer by continuing the culture. NSCs can differentiate into neurons, astrocytes and oligodendrocytes^19^. We confirmed the differentiation of NSCs into neurons and succeeded in recapitulating neurogenesis in a microfluidic platform. We observed relatively short neurites which are within 60 μm even on day 21, suggesting that cultured NSCs continued generating neurons for 21 days or some neurons stopped elongating their neurites in the beginning of the culture but still alive at the end of the culture. It is known that neural differentiation of NSCs is followed by glial differentiation^19^. Therefore, it is expected that glial differentiation will be induced by continuing the NSC culture in our culture model, which will lead to the construction of the 3D NVU structure in long-term culture.

BMECs and MSCs were cocultured in three different gel conditions, fibrin gel, fibrin-Matrigel mixed gel and fibrin-hyaluronan mixed gel. Firstly, we found that capillary-like structures with α-SMA-positive perivascular cells were constructed in these gel conditions. This is consistent with our previous study, which demonstrated that MSCs differentiate into α-SMA-positive cells in coculture with HUVECs^20^. Secondly, we found that fibrin and fibrin-Matrigel mixed gel promoted vascular formation more strongly than fibrin-hyaluronan mixed gel. In most previous angiogenesis studies, type I collagen or fibrin gels were used to culture ECs^21–24^. On the other hand, Matrigel is often used in 2D network formation assay^25^. Because Matrigel is composed of basement membrane proteins such as type IV collagen and laminin, which exist around mature vasculatures, Matrigel is not a suitable gel scaffold for inducing angiogenesis. In addition, we confirmed that BMECs failed to form vascular structures when they were cultured alone in fibrin-Matrigel mixed gel (Supplementary Fig. S2). However, BMECs successfully constructed capillary-like structures in fibrin-Matrigel mixed gel by culturing BMECs with MSCs. These results suggest that MSCs have a great potential to promote vascular formation. In our previous study we established a HUVEC-MSC coculture model to construct vascular networks in a microfluidic device^20^. Our recent study confirmed that MSCs not only differentiated into perivascular cells but also promoted capillary formation of HUVECs, leading to the construction of stable vascular networks covered by perivascular cells (unpublished data).

We hypothesized that hyaluronan would be suitable to construct brain vasculature because it is the main component of brain ECM^7^. However, interestingly, most cells migrated two-dimensionally along the bottom glass surface when they were cultured in fibrin-hyaluronan mixed gel. Some capillary-like structures were constructed in this condition but sheet-like structures were also constructed resulting from the two-dimensional cell migration, suggesting that hyaluronan is not suitable for constructing capillaries. Given that hyaluronan is one of the main components of brain ECM and angiogenesis is suppressed in mature normal brain, our data suggested that hyaluronan has some anti-angiogenic effects which lead to the stabilization of pre-existing vasculature. Addition of hyaluronan to hydrogel changed not only biochemical properties of the gel but also its mechanical properties. The stiffness of ECM may also affect the angiogenic process^26^. For example, we reported that the different stiffness of collagen gel led to the different vessel diameter and length in an angiogenic process^27,28^. Therefore, the further investigation will be needed in terms of the effect of biochemical and biomechanical cues on angiogenesis to establish a stable *in vitro* experimental model of NVU tissues.

Quantification of the constructed network length showed that the network length in fibrin gel and that in fibrin-Matrigel mixed gel had no significant difference. Therefore, we concluded that fibrin-Matrigel mixed gel is the optimal gel condition for culturing BMECs and MSCs as well as NSCs to induce vascular growth and neurite extension, respectively.

In the present study, we optimized hydrogel components in terms of 3D neurogenesis and angiogenesis, respectively. Based on the results, we combined both neural and vascular culture models and finally succeeded in constructing 3D neurovascular tissues with an optimized seeding condition of NSCs, BMECs and MSCs. A previous NVU culture model was based on a Transwell assay in which ECs and astrocytes and/or pericytes were cultured together^4,10^. These studies analyzed the integrity of endothelial barrier when they were cultured alone or cultured with astrocytes and/or pericytes. Another previous study presented a 3D NVU model which is composed of neurons, astrocytes and ECs in a microfluidic platform^29^. This study investigated endothelial barrier functions in NVU microenvironment. Therefore, this culture model is more physiologically relevant compared to *in vitro* BBB models with two-dimensional Transwell assays. In this culture model, ECs were cultured on a gel surface to form endothelial monolayer mimicking a blood vessel wall. On the other hand, in our study, we focused on the multi-step morphogenesis such as neurogenesis and angiogenesis in a microfluidic platform, resulting in the mimicry of the physiological NVU microenvironment. The present study is the first demonstration of a 3D neurovascular tissue culture model in which ECs constructed capillary-like structures through angiogenic processes while NSCs differentiated into neurons elongating neurites.

In our microfluidic culture model, especially in “NSC pre-seeding” condition, neurovascular tissues were successfully constructed on day 10 by inducing neurogenesis and angiogenesis simultaneously in an optimized 3D ECM hydrogel condition. However, in “same-day seeding” and “BMEC-MSC pre-seeding” conditions, the gel structure shrank within a few days from the BMEC-MSC side. This shrinkage of the gel can be explained by two factors. First, the gel structure was not strong enough against the traction force of BMECs and MSCs when they migrated into the gel. The other possibility is that BMECs or MSCs secreted MMPs to degrade the gel. Although there is still a possibility of further modification of the gel, the success in constructing neurovascular tissues including neurons and capillary-like structures is a critical step to achieve construction of NVU structures *in vitro*.

In conclusion, we established a microfluidic platform for constructing a 3D neurovascular tissue. This culture model enables us to culture NSCs and ECs three-dimensionally in a close proximity, which is mimicry of brain microenvironment composed of neural cells, capillary-like structures and ECM. In addition, we can monitor processes of neurogenesis, angiogenesis and the formation of neurovascular tissues by phase-contrast microscopy. Our microfluidic system has such advantages which cannot be achieved by conventional culture systems such as Transwell systems. This culture model can be applied not only for the investigation of brain functions which are performed through interactions among brain cells but also for drug screening studies as a therapeutic strategy. In the future study, we will investigate the differentiation of NSCs into glial cells such as astrocytes and oligodendrocytes, which are additional key elements constituting NVU microenvironments. A new *in vitro* NVU model which contains neurons, glial cells, blood vessels wrapped by pericytes and ECM proteins will be of great use.

## Materials and Methods

### Cell culture

NSCs were commercially obtained (Human Neural Stem Cell (H9 derived), GIBCO, Gaithersburg, MD, USA) and expanded in fibronectin-coated culture dishes by using the NSC medium kit (StemPro^®^ NSC SFM, GIBCO): Knockout DMEM/F12 with 2% StemPro^®^ Neural Supplement, 1% GlutaMAX, 20 ng/ml basic fibroblast growth factor, 20 ng/ml epidermal growth factor and 1% Antibiotic-Antimycotic (GIBCO). NSCs at passages 5 were used in this study.

BMECs were also commercially obtained (Human Brain Microvascular Endothelial Cells, Cell Systems Corporation, Kirkland, WA, USA) and cultured in CSC Complete medium (Cell Systems Corporation), which includes 10% serum. BMECs were expanded in fibronectin-coated culture dishes for no more than 7 passages.

Isolation of MSCs was described previously^20^. Briefly, MSCs were isolated from human bone marrow using LNGFR (CD271) and Thy-1 (CD90) surface markers. First, bone marrow mononuclear cells (Poietics^TM^; Lonza, Walkersville, MD, USA) were suspended at 1 to 5 × 10^7^ cells/ml in ice-cold Hank’s balanced salt solution supplemented with 2% fetal bovine serum (FBS), 10 mM HEPES and 1% penicillin/streptomycin. The cells were then stained for 30 min on ice with a monoclonal antibody. LNGFR-PE (Miltenyi Biotec, Bergisch Gladbach, Germany) and Thy-1-APC (BD Pharmingen, CA, USA) were used as antibodies. To eliminate dead cells from the flow cytometric analysis, propidium iodide (2 μg/ml) was used. Flow cytometric analysis and sorting were performed on a triple-laser MoFlo (Beckman Coulter, CA, USA) or FACSVantage SE (Becton Dickinson, Heidelberg, Germany).

MSCs were expanded in the MSC growth medium: Dulbecco’s Modified Eagle Medium with low glucose (DMEM, Invitrogen, Carlsbad, CA, USA) supplemented with 20% FBS, and 1% Antybiotic-Antimycotic (GIBCO). MSCs were used because they promote vascular formation and stabilize the structure of the vasculature^30^. MSCs at passages 6–8 were used in this study.

All three kinds of cells were cultured in a humidified 5% CO_2_ incubator at 37 °C. When coculturing BMECs and MSCs in a microfluidic device, CSC medium and MSC medium were mixed in the ratio of 1:1. In addition, all triculture experiments were performed by using NSC medium.

### Preparation of microfluidic devices

The fabrication process of the microfluidic device used in this study was described previously^31^. Briefly, the microfluidic device was made of poly-dimethylsiloxane (PDMS; Silgard 184, Dow Corning, Midland, MI, USA) and was produced by soft lithography with SU-8 patterned wafers. After peeling the cured PDMS from the SU-8 mold, it was cut into pieces and punched out to make media inlets and gel inlets. To form microchannels, each device was plasma-bonded with a coverglass (Fig. 1A). Microchannels were filled with 1 mg/ml poly-d-lysine solution (Sigma-Aldrich, St. Louis, MO, USA), and the devices were placed in a humidified 5% CO_2_ incubator at 37 °C for overnight. The devices were then rinsed twice with sterile deionized water and dried at 60 °C for 24 hours.

This microfluidic device has three channels. The central channel, which is filled with hydrogel, is sandwiched by two parallel microfluidic channels. The central channel has two gel-inlets which are 1.2 mm in diameter, and two microfluidic channels have two media-inlets which are 4.0 mm in diameter. The height of each channel is 135 μm. The width of the central channel is 750 μm or 1300 μm, and the width of microfluidic channels is 500 μm. The central channel whose width is 750 μm was used for triculture experiments to enhance potential interactions by the diffusion of growth factors secreted by both neural and vascular cells. We previously demonstrated that fluorescent dextran (MW 40 kD) injected in one channel can be transported across a gel region, whose width was 750 μm, by diffusion within 30 min^32^.

### Hydrogel filling and cell seeding in microfluidic devices

Fibrinogen was dissolved in phosphate buffered saline (PBS) to prepare pre-gel solution. This fibrinogen solution was injected into the central channel through the two gel-inlets immediately after thrombin (10 units/ml, Sigma-Aldrich) was added to the solution for starting gelation. A series of spaced PDMS posts works to prevent the pre-gel solution from leaking into adjacent microfluidic channels. The details in this process were described in a previous study^33^. Trapezoidal shapes were chosen for PDMS posts to achieve a uniform gel-fluidic channel interface^34^. Matrigel (Corning^®^, USA) or hyaluronan (HyStem^®^ Cell Culture Scaffold Kit, Sigma-Aldrich) was diluted with PBS and mixed with the fibrinogen solution on ice before thrombin was added to the pre-gel mixed solution. Concentrations of both fibrin and hyaluronan were 2 mg/ml, while concentration of Matrigel was 2, 4 or 8 mg/ml, depending on experiments. After the gel injection, devices were placed in a humidified 5% CO_2_ incubator at 37 °C for 30 minutes to complete polymerization of the pre-gel solutions. When injecting pre-gel solution, it was easily confined within the central channel because of surface tension between PDMS posts^31^. After gelation, microfluidic channels were filled with culture medium from media inlets. The microfluidic devices were kept in an incubator until use.

After dissociation from culture dishes using TrypLE (GIBCO), NSCs, BMECs or MSCs were suspended in each culture medium at the concentration of 1 × 10^6^ cells/ml. After aspirating the medium from reservoirs of a microfluidic device, 1 × 10^4^ cells of NSCs, BMECs or MSCs were seeded in each device. When seeding the cells, devices were tilted by 90° to allow them to adhere on the gel surface. Devices were then incubated for 30 min in a humidified 5% CO_2_ incubator at 37 °C. Culture medium was changed every day and phase-contrast images were taken every day to monitor the process of cell migration into 3D hydrogel. Immunofluorescence staining of the cells was performed as described below to visualize microvascular networks or neural networks.

### Immunofluorescence staining of cultured cells

For the imaging of constructed neural networks and vascular networks, cells were fixed with 4% paraformaldehyde for 15 min at room temperature and permeabilized with 0.1% Triton X-100 for 5 min. After permeabilization, the cells were treated with BlockAce (Dainippon Pharmaceutical, Japan) for 1 hour to block non-specific staining. The cells were then incubated at 4 °C overnight with primary antibodies, a mouse anti-α-SMA antibody (Sigma-Aldrich) for pericytes, a sheep anti-PECAM-1 antibody for BMECs, a mouse anti-Tuj-1 or anti-MAP2 antibodies for neurons. Thereafter, the cells were treated with secondary antibodies, Alexa Fluor 488-conjugated anti-mouse IgG (Invitrogen), Alexa Fluor 594-conjugated anti-rabbit IgG (Invitrogen) and Alexa Fluor 647-conjugated anti-rabbit IgG (Invitrogen), and incubated at room temperature for 2 hours. The cells were finally incubated with 4′,6-diamidino-2-phenylindole (DAPI; Invitrogen) for staining cell nucleus. The cells were rinsed with PBS between each step. Z-stack fluorescent images were taken by a confocal laser-scanning microscope (LSM700, Carl Zeiss, Germany). The 2D projection images were generated with the z-stack fluorescent images using ImageJ (National Institutes of Health, Bethesda, MD, USA).

### Quantification and statistical analysis of neurite length, vascular length, three-dimensionally located cell number, and degraded gel area

To quantify neurite length and vascular length, cells were fixed with 4% paraformaldehyde and stained as described above. After z-projection images were obtained with ImageJ (NIH), neurite length and vascular length were manually measured by tracing the neurites or vascular networks in immunofluorescence images. To quantify the number of three-dimensionally migrating cells, we counted cell nucleus which located at least 15-μm far from the top or bottom surface of a 3D hydrogel scaffold using ImageJ. This cell number was devided by the total cell number in the 3D hydrogel scaffold to calculate percentage of cells in 3D region. The area of the gel shrinkage was quantified using ImageJ by surrounding the area in each z-projection image. Experiments were repeated at least three times to confirm reproducibility of results. Data are presented as means ± s.e.m. A Student’s *t*-test was used to test for differences, which were considered statistically significant at *P* < 0.05.

## Electronic supplementary material

Supplementary Figures
