# Supplementary material for: Integration of neurogenesis and angiogenesis models for constructing a neurovascular tissue
Source: Sci Rep. 2017 Dec 11;7:17349. doi: 10.1038/s41598-017-17411-0 (PMC5725567; doi:10.1038/s41598-017-17411-0)
Supplement: Supplementary file 1 — Supplementary Figures [file 41598_2017_17411_MOESM1_ESM.doc]

# Supplementary Information

**Integration of neurogenesis and angiogenesis models for constructing a neurovascular tissue**

Hiroyuki Uwamori^1^, Takuya Higuchi^1^, Ken Arai^2^, Ryo Sudo^*1,3^

1. School of Integrated Design Engineering, Keio University, Japan

2. Neuroprotection Research Laboratory, Departments of Radiology and Neurology, Massachusetts General Hospital and Harvard Medical School, USA

3. Department of System Design Engineering, Keio University, Japan

# Supplementary Figures

**Figure S1. Formation of capillary-like structures in BMEC-MSC coculture in “same side” and “other side” seeding conditions.** A: Schematic illustrations of BMEC-MSC coculture. B, C: Representative phase-contrast images on days 0, 4 and 7, and corresponding immunofluorescence images of BMECs (PECAM-1, red), perivascular cells (α-SMA, green), and nuclei (DAPI, blue) on day 7. Scale bar, 100 µm. D: Quantitative analysis of network length. Data are shown as the mean ± s.e.m. (N=3, n≥9). n.s.: no significance.

**Figure S2. BMEC monoculture in fibrin-Matrigel mixed gel.** A: A schematic illustration of BMEC culture. B: Phase-contrast images on days 0, 1, 3, 5 and 7, and corresponding enlarged images on days 3, 5 and 7. Scale bar, 100 µm.
